# Examining the Relationship between Depression, Anxiety and Stress in Kidney Cancer Patients

**DOI:** 10.15586/jkcvhl.v9i1.199

**Published:** 2021-11-28

**Authors:** Türev Demirtaş, Zekeriya Temircan

**Affiliations:** 1 Department of Medical Ethics and History, Erciyes University School of Medicine, Merkez Kampüs Talas Yolu Melikgazi, Kayseri, Turkey;; 2 Department of Psychology, Kapadokya University, Yeni, Ürgüp, Nevs *¸*ehir, Turkey

**Keywords:** anxiety, depression, kidney cancer, patient, stress

## Abstract

Cancer of the kidney is one of the 10 most common cancers found globally. Overall, it is the fourth most common cancer in men and the eighth most common cancer in women. Many kidney cancer patients experience psychologic problems and reactions. The present study examined relationship between anxiety, depression, and perceived stress symptoms in kidney cancer patients. Cross-sectional data were obtained from the patients diagnosed with kidney cancer. All participants completed sociodemographic form, Hospital Anxiety and Depression form, and Perceived Stress Scale. Statistical analysis was exercised using the Student’s *t*-test, Chi-squared test (χ^2^), Fischer’s exact test, ANOVA, Mann–Whitney U test, and Kruskal–Wallis one-way variance analysis. A total of 250 patients participated in the study. The mean age was 57.4 years (SD 6.4, range = 25–76 years). The majority of patients were males (73%) and married (218). Anxiety symptoms were determined in 91.2% patients, depression symptoms in 87.2% patients, and perceived stress symptoms in 93.6% patients. The mean scores of Hospital Depression and Anxiety Scale (HADS)-Anxiety, HADS-Depression, and HADS-Perceived Stress were significantly different between age (P < 0.05), gender (P < 0.05), and income groups (P < 0.001). Kidney cancer patients showed poorer psychologic health. The overall levels of anxiety, depression, and perceived stress symptoms were higher among the studied kidney cancer patients. Findings of the current study could improve both psychologic well-being of patients and health-related quality of life.

## Introduction

Cancer of the kidney is one of the 10 most common diagnosed cancers worldwide. Overall, it is the fourth most common cancer in men and the eighth most common cancer in women (1). Studies have reported that prevalence of psychologic disorders and problems in kidney cancer patients is high (2). Owing to increasing lifestyle factors, such as age, gender, smoking, alcohol, obesity, and environmental and genetic factors, the patients are at higher risk of developing severe stress, depression, and anxiety (3). These factors and severe trauma have contributed to many psychologic problems in kidney cancer patients. Not only lifestyle factors but also stressful issues during the treatment have negative influence on patients’ quality of life. Recent studies have stated that increasing cancer cases are associated with different risk factors (4). However, it is important to investigate the effects of cancer in patients with many psychopathologies. It is well known that diagnosis and treatment of kidney cancer are very stressful issues for patients as well as family members. According to a study (5), two-thirds of patients who are mechanically ventilated experience high levels of stress and depression than their counterparts. The studies have also shown an association between patients’ prolonged mechanical ventilation and psychologic comorbidities such as depression, anxiety, and stress (6, 7). Another study reported that patients who had a longer treatment period experienced an increased risk of developing psychologic problems (8). There is growing evidence that cancer patients are at risk of developing psychologic problems such as stress, depression, and anxiety (9).

It is important to support cancer patients psychologically as well as psychosocially in order to achieve successful treatment process and improve quality of life. Although some attention has been paid to the increasing prevalence of psychologic disorders occurring in kidney cancer patients, there are limited data and research on relationship between stress, depression, and anxiety and kidney cancer. The results of these studies on kidney cancer patients are very limited in literature. Therefore, this study aimed to present fresh data by examining relationship between depression, anxiety, and stress in kidney cancer patients.

## Method

A total of 250 patients with diagnosed kidney cancer participated in the study. All participants completed sociodemographic questionnaire, Hospital Depression and Anxiety Scale (HADS), and Perceived Stress Scale (PSS). The sociodemographic form included patient’s personal information, including age, gender, marital status, education, income, occupation, chronic disease, health-related questions, and the type of surgical procedure being performed.

The HADS is commonly used to measure anxiety and depression levels of patients. It comprises 14 items and measures both anxiety (HADS-Anxiety) and depression (HADS-Depression) levels with an equal number of questions. Patients rank questions on a Likert scale, ranging from 0 to 3, and the subscale from 0 to 21. A total score of 8 or above was defined as an optimal cut-off score for level of comfort for both anxiety and depression. In a reliability study of the Turkish version of HADS, Cronbach’s alpha coefficient was found as 0.8525 for the anxiety subscale and 0.7784 for the depression subscale. The total correlation score of coefficient in the anxiety subscale was between 0.816 and –0.854, and that in the depression subscale was between 0.737 and –0.779.

The Perceived Stress Scale (PSS-10) which was validated and translated into Turkish by Eskin et al. (2013) was used to assess stress level. The Turkish version was also found to have adequate reliability and validity for measuring stress levels. The PSS-10 is about feelings and thoughts reflected by participants on their current life situations. This is a Likert scale to measure the level of stress (0 = never, 1 = almost never, 2 = sometimes, 3 = fairly often, and 4 = very often). Thus, the higher score shows higher level of stress. The scale correlates with depression and perception of poorer health quality.

### Statistical Analysis

Data were analyzed by SPSS statistical package program version 23. In statistical evaluation, Student’s *t*-test was used for quantitative variables, and numbers, percentage, mean value, and standard deviation were calculated. Chi-squared test (χ^2^), Fischer’s Exact test, ANOVA, Mann–Whitney U test, and Kruskal–Wallis one-way variance analysis were used. P < 0.05 was considered as statistically significant.

### Ethical Consideration

This study obtained ethical approval from the Erciyes University Medical School Ethic Committee, and Helsinki Declaration guidelines were followed during the study.

## Results

A total of 250 patients participated in the study. The mean age was 57.4 years (SD 6.4, range = 25–76 years). The majority of patients were males (73%) and married (218). More than half of the participants were aged between 25 and 50 years, and educated up to primary (36.8%) and secondary school levels. Nearly two-thirds of participants reported a monthly income of 2001–5000 Turkish Lira (TL). The majority of participants (66%) were not working or were retired, and were nonsmokers (79.2%). As for the health or clinical characteristics, 59.2% of the participants did not have surgery, 71.2% did not have cancer in the family, and 74% of them did not have other chronic diseases (Table 1).

**Table 1:** Socio-demographic characteristics of participants (*n* = 250).

		n	%
Age (years)	25–50	148	59.2
	50 and above	102	40.8
Gender	Male	184	73
	Female	66	27
Marital Status	Single	32	12.8
	Married	218	87.2
Education	Primary	92	36.8
	Secondary	67	26.8
	High School	48	19.2
	University and above	43	17.2
Income (TL)	<2000	71	28.4
	2001–5000	141	56.4
	>5001	38	15.2
Smoking	Yes	52	20.8
	No	198	79.2
Surgery	Yes	102	40.8
	No	148	59.2
Employment status	Working	85	34
	Not working or retired	165	66
Cancer in the family	Yes	72	28.8
	No	178	71.2
Other chronic diseases	Yes	185	74
	No	65	26

TL: Turkish Lira

The mean scores of the anxiety and depression subscales were 1.9 (SD = 2.9) and 1.8 (SD = 2.5), respectively. The mean score of the distress subscale was 2.1 (SD = 2.8). HADS-Anxiety symptoms in the study were found in 91.2% patients; 98 (39.2%) participants had mild, 99 (39.6%) had moderate, 31 (12.4%) had severe, and 22 (8.8%) had no anxiety symptoms. HADS-Depression symptoms in the study were found in 87.2% patients; 87 (34.8%) participants had mild, 101 (40.4%) had moderate, 30 (12%) had severe, and 32 (12.8%) had no depression symptoms. PSS-Stress symptoms in the study were found in 93.6% patients, with 176 (69.6%) participants having moderate, 60 (24%) having high, and 14 (6.4) having low or no stress symptoms.

Table 2 presents comparison of the mean scores and total sample numbers of HADS-Anxiety, HADS-Depression, PSS-Stress, and sociodemographic variables. The mean scores of HADS-Anxiety (independent *t*-test (*t*) = –1.87, P = 0.01), HADS-Depression (*t* = –1.77, P = 0.04), and PSS-Stress (*t* = 2.54, P = 0.02) were significantly different between age groups. The mean scores of HADS-Anxiety (*t* = 0.91, P = 0.01), HADS-Depression (*t* = 1.02, P = 0.02), and PSS-Stress (*t* = 0.067, P = 0.02) were significantly different between genders. Likewise, mean scores of HADS-Anxiety (*t* = 2.87, P = 0.01), HADS-Depression (*t* = 2.38, P = 0.02), and PSS-Stress (*t* = 3.12, P = 0.01) were significantly different between income groups. Participants aged ≥50 years, having primary and above education, and were not employed or retired reported significantly poor psychologic well-being than their younger, higher educated, and employed counterparts. Females reported poorer HADS-Anxiety and HADS-Depression scores as compared to males; however, males reported poorer PSS-Stress scores than females. Single participants reported low mean scores in HADS-Anxiety, HADS-Depression, and PSS-Stress scales as compared to married participants. In addition, smokers and those with other chronic diseases reported poorer HADS-Anxiety, HADS-Depression, and PSS-Stress scores as compared to nonsmokers and participants not having other chronic diseases. Finally, participants having cancer patients in their family reported high mean scores in HADS-Anxiety, HADS-Depression, and PSS-Stress than their counterparts having no cancer patients in their family (Table 3).

**Table 2:** Anxiety, depression, and perceived stress in kidney cancer patients (*n* = 250).

	n	%
HADS-Anxiety		
No anxiety	22	8.8
Mild	98	39.2
Moderate	99	39.6
Severe	31	12.4
HADS-Depression		
No depression	32	12.8
Mild	87	34.8
Moderate	101	40.4
Severe	30	12
PSS-Stress		
Low	14	6.4
Moderate	176	69.6
High	60	24

**Table 3:** Comparison of mean scores of HADS-Anxiety, HADS-Depression, PSS-Stress, and socio-demographic characteristics (*n* = 250).

	n	HADS-Anxiety Mean (SD)	HADS-Depression Mean (SD)	PSS-Stress Mean (SD)
Age (years)				
25_50	148	1.4 (1.8)	1.2 (2.0)	1.17 (2.34)
50 and above	102	2.1 (3.2)	2.0 (2.8)	2.1 (2.98)
t		–1.87	–1.77	2.54
P-value		0.01	0.04	0.02
Gender				
Male	184	1.7 (2.8)	1.7 (2.5)	2.1 (2.8)
Female	66	4.0 (4.8)	2.8 (2.6)	1.8 (2.4)
t		0.91	1.02	0.067
P-value		0.01	0.02	0.02
Marital Status				
Single	32	2.2 (3.3)	2.1 (2.8)	2.6 (3.2)
Married	218	1.6 (2.7)	1.5 (1.4)	1.8 (2.6)
t		0.41	0.43	0.87
P-value		0.672	0.322	0.812
Education				
Primary	92	3.8 (2.6)	2.4 (2.2)	2.9 (4.4)
Secondary	67	0.9 (1.2)	1.0 (1.8)	1.5 (2.2)
High School	48	1.7 (2.6)	1.1 (1.6)	1.9 (2.7)
University and above	43	2.0 (3.5)	2.2 (2.6)	2.4 (3.4)
F		0.29	0.09	3.12
P-value		0.18	0.91	0.01*
Income (TL)				
<2000	71	2.2 (1.4)	2.6 (1.7)	2.2 (1.6)
2001–5000	141	2.0 (1.5)	2.4 (1.5)	2.1 (1.6)
>5001	38	2.6 (1.4)	2.2 (1.7)	2.3 (1.4)
t		2.87	2.38	3.12
P-value		<0.01**	0.02*	0.01
Smoking				
Yes	52	5.1 (5.2)	2.6 (1.5)	1.8 (1.8)
No	198	3.0 (4.9)	2.1 (1.2)	1.4 (2.2)
t		–0.45	–0.344	–1.91
P-value		0.29	0.417	0.812
Surgery				
Yes	102	3.5 (4.8)	3.2 (4.2)	2.8 (4.1)
No	148	3.2 (4.4)	2.7 (3.8)	3.0 (5.2)
t		0.718	0.578	0.612
P-value		0.772	0.812	0.433
Employment Status				
Working	85	2.0 (3.2)	1.8 (3.2)	1.6 (2.0)
Not working or retired	165	3.1 (4.4)	2.5 (3.6)	1.8 (2.8)
t		–0.221	–0.239	–0.518
P-value		0.918	0.377	0.617
Cancer in the family				
Yes	72	3.9 (5.6)	3.2 (3.0)	2.7 (4.1)
No	178	2.7 (1.5)	1.6 (2.5)	1.5 (2.1)
t		0.59	0.678	0.805
P-value		0.122	0.433	0.718
Other chronic diseases				
Yes	185	4.4 (6.2)	4.1 (5.8)	3.8 (2.6)
No	65	3.5 (4.0)	2.6 (1.4)	2.8 (3.2)
t		0.481	0.756	0.32
P-value		0.245	0.456	0.978

*t*: Independent *t*-test; F: One way ANOVA; TL: Turkish Lira.

* Significant level at 0.05; ^**^significant level at 0.01.

## Discussion

The results of the current study indicate that psychologic disorders of anxiety, depression, and stress, with their subscales, appeared in nearly 90% of patients with kidney cancer. Kidney cancer patients may experience any level of anxiety, depression, and distress at any stage of their disease after diagnosis. It is assumed that cancer is a serious disease and causes many psychologic problems. In the current study, HADS-Anxiety, HADS-Depression, and PSS-Stress scores were used to screen kidney cancer patients. The prevalence of anxiety, depression, and perceived stress symptoms were high in these patients. Significantly greater anxiety, depression, and perceived stress symptoms were specifically reported in relation to age, gender, income, and sociodemographic variables.

**Figure 1:**
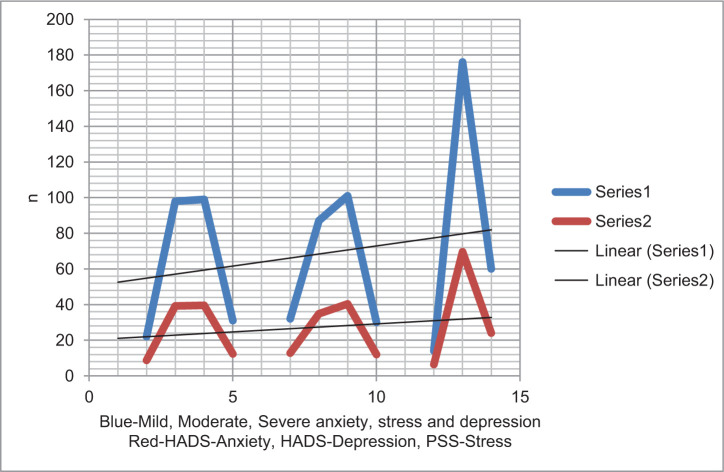
Distribution of anxiety, depression, and perceived stress in kidney cancer patients (*n* = 250).

An increase in psychiatric morbidity has been reported in cancer patients, and the prevalence of psychiatric diseases has been found to vary in a wide range of 9–60% (10). In the current study, the percentage of anxiety, depression, and perceived stress in kidney cancer patients was considerably higher compared to the data available in literature. It was found in literature that most patients remained anxious due to the process of treatment, stage of cancer, thoughts of death, extended hospital stay, and other health complications (11). Li and Wang reported that 77.6% of Chinese kidney cancer patients had depressive symptoms and 68.3% of them had anxiety symptoms because of series of negative emotions, confronting progressive illness, and longer treatment period (12). Supportively, Cohen et al. indicated that 23% of kidney cancer patients in the United States experienced depressive symptoms (13).

A systematic observational study conducted on the prevalence of psychologic problems in cancer patients showed that cancer diagnosis and treatment interact with the level of anxiety and psychologic state of the patient, thereby negatively affecting patient’s current illness, fight with the disease, compliance with the treatment, and quality of life (14). Henningsohn et al. reported that 35% of cancer patients had depressive symptoms and 20% of them had anxiety symptoms after surgery (15). Relatively, Ficarra et al. showed that 22% of the patients with urologic malignancy experienced depressive symptoms and 14% of them had anxiety symptoms (16). Another study (17) has reported that age is associated with the risk for anxiety and distress, as younger patients tend to worry more about cancer and the manner of treatment. Generally, young and middle-aged cancer patients, females, and those receiving chemotherapy have higher levels of anxiety and distress. The study reported that the highest rate of anxiety and distress was found in patients aged 18–40 years. Findings of the current study are consistent with the above-mentioned results, as age and gender are linked to anxiety, depression, and perceived stress in kidney cancer patients.

Income was identified as one of three predictors of higher risk of anxiety, depression, and perceived stress in kidney cancer patients. The study conducted by Sanfilippo et al. revealed that cancer patients from low-income group experience more psychologic problems because of lack of access to medical care, medicines, and not getting enough treatment after diagnosis (18). Inability to access medical resources is associated with more psychologic problems in these patients. Findings of the current study are consistent with those found in literature, as income is considered as one of the predictors in kidney cancer patients liked to anxiety, depression, and perceived stress symptoms. Recent studies have indicated that low education and income and lack of psychologic resilience are the risk factors for depressive and anxiety symptoms (19). Another study has reported that individuals affected by cancer face decreased ability to keep their lives under control, have increased dependency on others, with deteriorating balance in family, work, and social life (20). Thus, cancer patients experience greater level of anxiety, depression, distress, and other psychosocial problems. Therefore, at social level, concerns may arise about their relationships with family members, healthcare professionals, and social network. Although participants in the current study demonstrated a higher risk of anxiety, depression, and perceived stress symptoms, prior studies have reported that patients with breast, prostate, and lung cancers also demonstrate having these psychologic problems during the process of illness (21, 22).

## Conclusion

This is the first study to examine relationships of psychologic anxiety, depression, and stress in kidney cancer patients in Turkey. The results of this study provided knowledge on psychologic problems such as anxiety, depression, and perceived stress symptoms in kidney cancer patients. Our findings revealed that age, gender, and income mainly affected depressive, anxiety, and stress symptoms in kidney cancer patients. Results incurred in the present study were in accordance with literature findings on kidney cancer and psychologic problems. Hence, it is important for hospitals to create feasible and positive strategies to promote psychologic resilience of kidney cancer patients. It is also important to improve their emotional and psychologic well-being as well as increase their quality of life.

## Implications

This is the first study in Turkey to evaluate psychologic problems such as anxiety, depression, and stress in kidney cancer patients. Findings of this study have practical implications to cope with psychologic problems. The findings revealed that kidney cancer patients not only suffer from cancer but also from psychologic problems. Further studies must focus at exploring whether psychologic interventions and cancer-related education could help patients to cope with cancer and its psychologic symptoms.
